# ‘They didnae tell you nothin’: The Failings of Sex Education, Antenatal Care, and Welfare Bureaucracies in Glasgow, *c*. 1970s–2000s

**DOI:** 10.1093/tcbh/hwac009

**Published:** 2022-03-31

**Authors:** Janet Greenlees

**Affiliations:** Glasgow Caledonian University, UK

## Abstract

Utilizing group oral histories from nineteen women who were pregnant and living in areas of social and economic deprivation in Glasgow, Scotland, between the late 1970s and early 2000s, this article analyses the difficulties the women faced in accessing information about pregnancy and welfare entitlements. It reveals a disconnect between women’s knowledge about reproduction and maternal health and welfare benefits and the political initiatives designed to improve antenatal care and pregnancy outcomes in Britain since the 1980 Short Report. This divide was widened by a broader Scottish culture of reticence around sex education and the ongoing moral influence of the churches. The article clarifies the class-blind English arguments within the patient consumer model that was promoted since the 1960s. It demonstrates how marginal groups were ill equipped to participate as patient consumers, either individually or as a collective group. More broadly, this article gives voice to an underrepresented group and highlights how these women utilized adaptive decision-making to navigate their pregnancy journeys in a society with uneven maternity and welfare provision and inhibitions about sex education. By highlighting the realities of marginality and lived experiences, it adds nuance to conventional welfare and policy histories.

In 2017, a participant in a group discussing pregnancy experiences while living on low incomes in late twentieth-century Glasgow, Scotland, exclaimed ‘They didnae tell you nothin!’ She was referring to her high school teachers and the midwives and doctors she had encountered who had provided little information about pregnancy or associated welfare benefits. Indeed, the nineteen participants in this oral history project confirmed the lack of health and welfare information about their pregnancies from educators and healthcare professionals, demonstrating the failings of associated British government policy and medical care. By contextualizing and analysing this gap in Glasgow, this article uses lived experiences of poverty to challenge conventional welfare and policy histories. In so doing, it gives voice to low-income women’s experiences of maternity by highlighting their struggles to access information about pregnancy and welfare entitlements in late twentieth-century Scotland.

Scottish health and welfare provision has been administratively, as opposed to politically, devolved from Britain since well before the 1947 National Health Service (Scotland) Act. Also, Scotland has an historically high proportion of its population living in low-income households, or households with an income before housing costs of less than 60 per cent of the country’s median income in that year. Within Scotland, per capita, the city of Glasgow has historically had the highest concentration of poverty, with between 45 and 47 per cent of households affected in 2000.^1^ Such persistent high levels of poverty bring with it subjection to the whims of welfare and the state through class, while also having a detrimental impact on health. Indeed, the people of Glasgow have the lowest life expectancy and poorest health compared to the rest of the country, the UK and Europe, which is often referred to as the ‘Glasgow effect’. However, poverty’s impact on health and particularly maternity is complex and alone, does not account for Glasgow’s health disparities.^2^ Lived experiences of maternity can reveal both the successes and failures of health and welfare provision because they are intimately connected with all women’s experiences of motherhood. The testimonies explored here highlight how women’s understandings of pregnancy were shaped by their experiences of poverty and medical and welfare encounters that were distant from political and medical rhetoric and initiatives and which reflected the Scottish reticence towards sex education.

British public interest in pregnancy and childbirth and associated social policy grew during the latter quarter of the twentieth century and stemmed from the social movements of the 1970s, particularly the women’s movement.^3^ There was an increased focus on women’s healthcare and the monitoring of pregnancies where sociologist Ann Oakley found that by the mid-1970s, the average number of antenatal visits was thirteen.^4^ Alongside this ran anti-medicalization and anti–medical-technology lobbies that had originated during the 1960s.^5^ Patients were increasingly becoming political actors in maternity debates.^6^ Public concern was mounting that babies were unnecessarily dying or suffering permanent damage, while infant mortality rates in England and Wales were falling more slowly than in many other developed countries. These concerns contributed to the Report of the Social Services Committee on Perinatal and Neonatal Mortality, or Short Report, published in December 1980. Most obstetricians and neonatologists hailed this as almost a New Testament. Among the 152 recommendations was that ‘The whole concept of antenatal care must be improved, probably starting with health education in the schools for boys and girls.’^7^ Moreover, antenatal appointments should provide the expectant mother with information, advice, and reassurance alongside health checks. The Scottish Health Education Group (SHEG) agreed.^8^ Since then, government and medical efforts have prioritized improving patient interactional skills among trainee doctors, rather than improving the style and content of the information provided to expectant mothers.^9^ This left the nature and content of clinical encounters dependent on individual doctors and midwives.

Indeed, the Short Report questioned ‘what exactly antenatal care consists of’ and there was no single or easy answer in Britain. By the 1980s, a pregnant Scotswoman could expect at least three antenatal appointments to check the mother’s blood pressure and weight gain and that the baby was growing well. Ultrasound scans were becoming routine to view the foetus and determine gestational age. However, not all women were convinced of the benefits of increased monitoring during pregnancy, particularly when consultations might last only between 3 and 6 min.^10^ By 1983, Scottish maternity services were supposed to be integrated, with midwives conducting the majority of prenatal checks. However, depending where a woman lived she might see a midwife, a General Practioner (GP), or both.^11^ This contributed to patient confusion about providers and their responsibilities, detracting from the continuity and consistency of both pregnancy care and information. While our interviewees’ antenatal experiences varied in number and location, there was no noticeable norm. The women lived in different parts of Glasgow where provision varied.

The lack of antenatal reform after the Short Report is unsurprising given that no funds had been allocated to implement any of the recommendations. Therefore, school health education changed little and most women were left to source their own antenatal information rather than become healthcare consumers. Alex Mold has described how towards the end of the twentieth century, successive governments promoted the ideal of the British patient as a healthcare consumer with associated behavioural expectations.^12^ This relates to the growing belief in a patient’s (and consumer’s) right to know and the doctor’s role in communication, as well as debates about relationships between choice and equity.^13^ However, notions of consumerism and citizenship are varied and constantly in flux.^14^ Hence, the changing expectations of low-income pregnant women as patient consumers posed challenges because the ability of a consumer to navigate the healthcare system was dependent, among other things, on being able to access relevant and accurate information. This Scottish study reinforces Matthews and Hastings argument that the interplay between English middle-class service-users and healthcare providers influenced the development of public services to meet middle-class needs rather than working-class realities.^15^

British medical debates about maternity care have subsumed antenatal care so that low-income mothers rarely feature.^16^ Oakley argues that British antenatal provision was a version of the medical imperialism thesis, whereby doctors continually seek to expand their authority.^17^ This argument has been mirrored elsewhere,^18^ with maternity histories highlighting inter-professional rivalries and middle-class women’s struggle for greater control over their pregnant bodies.^19^ Such arguments embody certain middle-class values and expectations about the centrality of the medical control of pregnancy and childbirth and what comprised a family. While historians including Hilary Marland, Lara Marks, and Susan Williams have investigated women’s agency as mothers, their research has primarily examined voluntary groups, political pressure groups and organizations, most of which, like the National Birthday Trust Fund, comprised middle-class members.^20^ Poorer women entered debates almost like objects as Lindsey Earner-Byrne found in twentieth-century Dublin, where religious notions of morality shaped maternal medicine.^21^ Nevertheless, whether criticizing medical systems, as Oakley did, or adopting a more nuanced account like Jane Lewis, Jan Williams, Angela Davis, and Alison Nuttall, scholars have suggested that medical care has the potential to change behaviours.^22^ People, here expectant mothers, are expected to behave in certain ways and to attend clinics at the convenience and behest of the healthcare provider.^23^ Such behavioural expectations for healthcare participation have associated financial constraints and imply power relationships.

In any country, state and medical expectations of maternal behaviour place pressures on the economically disadvantaged through travel costs, potential time off work, and simply by forcing women to join the unfamiliar, wealthier environment of healthcare. Since the Second World War, experts on child development, including John Bowlby, Donald Winnicott, Benjamin Spock, and Penelope Leach, sought to empower women to take more control of their pregnancy and infant care. Their literature targeted an educated readership and was criticized by the middle-class women’s movement as being sexist and patriarchal, while fuelling their desire for greater control over their own bodies.^24^ Although not all women would have read these literatures, by the 1980s the trend in maternity education was towards ‘responsibilization’, or the loading onto women responsibilities for self-educating about pregnancy, childbirth, and infant welfare and managing their own health. By design, such expectations excluded poorer women from becoming patient consumers. By the 1990s the government had become the core force in shaping the patient as a consumer and, as Peter Matthews discovered, public services had a generally favourable pre-disposition towards middle-class needs and reflected class-hierarchies.^25^ Both in Scotland and in Yorkshire, England, three-quarters of poor women reportedly learnt nothing from their antenatal check-ups and were skeptical about the value of routine antenatal care.^26^ Little had changed by the end of the century. In 2001 Gross and Pattison found the tone of the available pregnancy advice literature to be ‘patronizing’ and ‘hectoring’ and to contain contradictory and banal messages.^27^ Within a middle-class environment of hospital, healthcare, and antenatal classes and where the patient was expected to fit in with the hospitals’ needs and requirements,^28^ the needs of the pregnant women who did not fit this norm could be easily overlooked. This article suggests they were.

By analysing the experiences of women who had been living in one of the most deprived 10 per cent of all Scottish communities when they delivered their first baby in Glasgow between 1969 and the early 2000s, this article gives voice to women’s beliefs, values, and experiences of motherhood while living on a low income. It contributes to the growing body of oral histories about sexuality and contraception, including Elizabeth Roberts and Lucinda Beier’s work on family social changes in early twentieth-century Lancashire, Kate Fisher’s and Szreter and Fisher’s candid work about intimate life in England and Wales between 1918 and 1963.^29^ Some of our interviewees became pregnant outside of wedlock and their narratives help to map their emotions and experiences in Scotland, adding to Pat Thane and Tanya Evans analysis of the ‘real lives of unmarried mothers’ in twentieth-century England, but which relied heavily on printed sources.^30^ Over 100 years after poor women wrote to Margaret Llewelyn Davies advocating for improved maternity care across Britain in a 1915 Women’s Co-operative Guild collection of letters, our interviewees make similar requests.^31^

These women’s narratives draw on Scotland’s oral history tradition to help make sense and meaning of their experiences and to bear witness to them.^32^ Together with the charity Poverty Alliance,^33^ four groups of women were recruited from areas of multiple deprivations within Glasgow. These women had resided in these or nearby neighbourhoods most of their lives, never joining the middle-classes. The groups were self-selecting in that all the women volunteered to be interviewed. The focus groups were conducted by the author and Fiona McHardy, the Research Officer for Poverty Alliance and took place within the women’s local community for participant convenience and familiarity. The group interview format supported gathering a broad range of information about personal and collective experiences, feelings, perceptions, and opinions. They also helped to informally jog memories and assist a more natural flow to the conversation. We also recognized that the women might find group interviews to be more enjoyable, making recruitment easier, and helping to create a relaxed environment for discussions.

Prior to each interview, we collected written biographical information from each woman, along with simple facts about her pregnancies, including: the number of children conceived with dates and locations of birth; any multiple births; which healthcare professional was their first contact during pregnancy and roughly, at how many weeks pregnant was that first visit; approximately how many antenatal appointments were attended; and their memories about both maternity care and the providers. The form utilized tick-boxes, with images and cartoons to aid recollection, ease any stressful memories, and to accommodate all education levels. This strategy enabled the interviews to focus solely on women’s lived experiences of pregnancy on low incomes and allow individuals to interpret their own stories. The semi-structured interviews traced the women’s pregnancy journeys from their knowledge about pregnancy and childbirth, through when they first realized they were pregnant, their experiences at healthcare appointments, who provided antenatal care and where, the extent to which financial concerns influenced behaviours and actions, and their experiences of childbirth and postnatal care. Unstructured, open-ended interviews enabled respondents to present their stories at length, independent of the interviewers’ original analytical agenda. As is widely acknowledged with oral histories, ‘when the survey subject is complex or emotional, it may be that the greater flexibility of an informal approach succeeds better than set questions in getting to the heart of the respondent's opinion’.^34^ We also recognized that experiences of pregnancy and poverty are personal and individual and did not push any participant to share if she did not want to. While this left the information uneven, the lack of pressure to contribute made the women more comfortable sharing selected experiences. We also noticed how the women’s openness increased during the course of each interview. For many women, these interviews marked the first time they had discussed their pregnancy experiences.

The responsibilities of pregnancy and motherhood were clearly gendered. However, the distinct absence of fathers must be noted. Interviewees rarely mentioned the fathers and usually only in relation to particular events. These included providing practical assistance, such as taking the woman to hospital when labour started or accompanying mother and baby home, and violence towards and/or abandonment of their pregnant partner. Fathers did not attend antenatal appointments. While probing fathers’ involvement was outside this project’s scope, their noticeable absence suggests whether from necessity or gendered assumptions surrounding pregnancy, mothers were left to secure their own information and advice. As had their forebears, women relied on female support networks,^35^ and increasingly, antenatal appointments.

By teasing out the antenatal knowledge and experiences of pregnant Glasgow women living on low incomes in the decades around 2000, this article clarifies the class bias entwined within the patient–consumer model promoted by the British government since the 1960s. Not all citizens enjoyed the increasing right to information, voice, and choice. Moreover, the framing of the patient consumer as a political construct can overshadow the large numbers of patients who participate in healthcare but who fall through cracks in government and social ideals about what comprises a patient consumer. Alex Mold has demonstrated how increasing choice in a collective system only achieved partial success. The middle-class consumer groups who led the campaigns for patient autonomy and representation ignored working-class needs.^36^ This article demonstrates how low-income Glaswegian women failed to, or were unable to, conform to expectations about their patient-consumer role. To that end, a number of issues are examined in turn: women’s reproductive knowledge and Scottish reticence about sex education; the multiple pregnancy messages women received; the failure of medical professionals to communicate in a clear and approachable manner; and, the paradox surrounding welfare benefits. Throughout, it highlights how women utilized adaptive decision-making or a variety of strategies to make judgements and choices for navigating the complexities of their pregnancy journeys.

## Scottish Moral Dynamics and Reticence about Sex Education

During the 1980s and 1990s, sex education was fiercely debated in the UK. Jane Lewis and Trudie Knijin and others have convincingly argued how the adversarial nature of sex education in England and Wales resulted in messages that lacked coherence. In turn, these were reflected in the classroom. Simon Blake, then director of the Sex Education Forum, explained that it was not clear whether the aim of sex education was to ‘prevent teen pregnancies, make teenagers understand their bodies, or contribute to personal and social development’.^37^ Education policy was permeated by morality and the values of family life.^38^ Nevertheless, after the 1944 Education Act, school sex education gradually led to a more publicly acknowledged respectable discourse about the body’s reproductive functions, including menstruation.^39^ Indeed, Szreter and Fisher found that the post-Second World War baby boom generation were not as sexually ignorant as initially believed, although asserting their ignorance provided women with a certain amount of respectability.^40^

In Scotland, Christian morality has long influenced public versus private debates concerning sex, motherhood and sex education and was reflected in policy.^41^ During the 1970s, the Moral Welfare Committee of the Church of Scotland agreed that the sex education curriculum must include moral education and be ‘reflective of Christian values’, including chastity and social responsibility.^42^ Taught separately, sex education could corrupt the young and innocent and promote permissiveness. Instead, safe sex could only be mentioned in relation to marriage.^43^ With similar timing, the Roman Catholic Church in Scotland developed its own national syllabus for sex education which also placed sexual matters within a framework of ‘moral and ethical enlightenment’.^44^ However, the devolved nature of Scottish government meant that unlike in England and Wales, the content of sex education was locally determined. While the extent and breadth of sex education varied across Scotland, during the 1970s only 40 per cent of primary schools taught lessons that included birth, reproduction, or menstruation.^45^ Similar to sex education, Scottish women’s access to reproductive information and strategies, including contraception and abortion, was primarily determined by local rather than national forces.^46^ There was little contraceptive advice available for unmarried women.^47^ Moreover, political and social campaigns had sought to exclude Scotland from the 1967 Abortion Act on grounds that it failed to correspond with the needs or values of Scottish society. Instead, the existing medical discretion surrounding terminations sufficed.^48^ Across Scotland reproductive information remained patchy, basic, and shaped by the socially conservative teachings of the churches. In Glasgow, strong sectarian tendencies made sex education particularly contentious.

During the 1980s, the Scottish Education Department refused to coordinate a consistent national provision of school sex education, partly because of ongoing resistance to such an initiative from parents, teachers, educational authorities, and civic leaders.^49^ When the 1988 National Curriculum made the biological aspects of reproduction part of the science curriculum, this did not extend to Scotland. Furthermore, biology and sexuality were separated.^50^ The 1986 Education Act allowed schools to not provide sex education and for parents to withdraw their child from an offered class. While the subsequent 1993 Education Act made sex education compulsory in secondary schools, parents could still withdraw their child from any or all lessons. Although the 1980s and 1990s Take Care AIDS educational initiative did much to draw discussions about sex into the open in England and Wales, as Angela Davis found for Oxfordshire, public discussions about sex and reproduction were a gradual change.^51^ Moreover, these initiatives failed to have a strong influence in Scotland. As one witness to the House of Commons Social Services Committee on AIDS testified in 1987, there remained a ‘moral infrastructure of health and social education in Scottish schools which carrie[d] a sex education programme to which AIDS [*could*] be added’.^52^ The core message of health education programmes remained ‘abstinence’ except in marriage. Even in 2017 the Scottish government refused to make sex education compulsory in state schools and parents could and did still request that their children not attend these classes.^53^

This was the context in which our interviewees grew up. When asked about family discussions about reproductive biology and women’s health or school sex education, most women recalled receiving little information. Concerning puberty, one woman remembered how ‘my mother never told me about my period or anything, you know’. A fellow interviewee continued that when her period started, she screamed, adding, ‘I was the same, I was the same. I thought I was dyin.’ When she told her mother, the latter simply laughed, and replied, ‘my wee girl’s a wee woman now’.^54^ Starting her period marked the change from childhood to adulthood. However, family inhibitions about discussing sex left ignored the associated implications and responsibilities. Alice remembered after telling her Granny that she was bleeding, her Granny simply replied, ‘put them on, you’, white things, horrible, …but nae explanation, eh, what was happenin’, and away to yer bed…’.^55^ Indeed, all but two of the nineteen women interviewed confirmed that neither their mothers nor grandmothers openly discussed periods or reproduction and whose inhibitions reflected their upbringing. The longevity of Scottish conservatism surrounding discussing sex and sexuality reflected consensus within certain Glasgow communities.

Alice, who was born in 1968 and had her first baby in 1996, remembered the adult’s widespread reluctance to discuss sex with teenagers. ‘It [reproduction] was a taboo subject, I think.’ Others agreed that reproduction ‘was never spoke about really’. ‘[Y]our sex ed classes at school were rubbish, you know what I mean, they didnae tell you nothin.’^56^ Mary remembered how ‘…when I was at school, you were told that the baby’s seed was in cabbages, and I was fully pregnant…. I think they just said anything, if you asked something, they were so…they were all tensed up.’ Mhairi remembered being in upper primary school and asking her mother, ‘How does a baby get in your tummy, ye gonnae show me Mummy? It’s like they just didnae know what to say.’ Parents deflected questions to each other. ‘Ask your Dad’… or ‘ask your Mum.’ Most women remembered the sole parental advice was ‘Keep yerself clean and keep away fae boys’, reflecting social expectations concerning female respectability and behaviour but without the associated knowledge and understanding.^57^

The women’s reproductive ignorance but desire for accurate information reflects that of their forebears, including those who wrote to the Women’s Cooperative in 1915, and many of Fisher’s and Davis’s interviewees about sex and maternity from the 1920s through the 1980s in England and Wales.^58^ Yet, the British narrative is inconsistent. Beier found Lancashire working-class attitudes towards sex became increasingly open among those born after 1930 and Szreter and Fisher have persuasively challenged the picture of repression and ignorance in English sex cultures before the 1960s.^59^ In Scotland, the broader British trend towards greater openness surrounding sex and reproduction was overshadowed by the strength of local cultures. Indeed, Andrew Blaikie found continuous high rates of illegitimacy in Northeast and Southwest Scotland across the nineteenth and twentieth centuries which reflected a less condemnatory attitude towards unwed motherhood than was found in Glasgow.^60^

Glasgow social attitudes towards sex were entwined with women’s sexual and moral behaviour. When in 1975 Jane fell pregnant at age sixteen, she remembers:

When I told my Mum I was pregnant, she went and told his Mum and then before I knew it, they had the date for me to get married…. They arranged it, aye, I didn’t even have a say, so it happened, I didnae have a say. Just, ahh, I cannae have a child oot o’ wedlock.^61^

The need to avoid family shame excluded Jane from key decisions about her life and reflected what Amy Shalet found for the USA in the 1990s—namely, that many parents were not prepared to acknowledge the degree of change that had occurred in family formation and teenage sexual behaviour. Parental control, without family negotiation, dominated relations.^62^ Ten years later cohabiting was more acceptable in Glasgow and across Scotland, so long as the woman did not become pregnant.^63^ When women like Amy became pregnant in 1984 at age twenty-four, having lived with her partner for several years, their parents expected marriage. Amy’s Mum told her partner: ‘I hope you’re gonnae do the decent thing and marry her.’^64^ Even in 2000, the centrality of traditional moral attitudes towards pregnancy and marriage remained. The 2000 Scottish Social Attitudes Survey revealed that the majority of Scots considered marriage to be the best kind of relationship; that men and women wanting a family should marry; and that Scotland had a problem with teenage pregnancy.^65^ Young women were caught in the precarious position between ignorance and societal expectations. In a society with little respect for difference, many women were left confused about what to do and where to turn for help, advice, and support.

By the mid-1980s, abortions were becoming more socially acceptable in Scotland and offered an alternative to unwed-motherhood, yet none of our interviewees mentioned having or wanting a termination. Lilly described how her boyfriend wanted her to have an abortion when she fell pregnant at age eighteen. She recalled, ‘I remember walkin’ roond Asda and sain’ to my Mum, ‘I’m pregnant’, and she just [gasps], her mouth just dropped, you know, and then when I told the Dad, he just says, ‘best thing I think that you could do is have an abortion’.^66^ After he left, Lilly raised her son by herself. Although she did not elaborate or express any religious convictions, to Lilly, abortion was not an option. While potentially relating to a religious upbringing, her conviction may also reflect the moral conservatism that remained entrenched within Scottish civil society during the 1980s when many of our interviewees first became pregnant. Moreover, when Lilly was growing up during the 1970s, the British desire for greater personal autonomy and self-determination developed and manifested itself in multiple forms, including untraditional family structures.^67^ During the 1980s, the importance of individualism grew and was reinforced by Prime Minister Margaret Thatcher. She sincerely believed that poverty was caused by personal rather than social failings and advocated that families, not the state, should support individuals in times of need.^68^ It is unclear the extent to which such environments influenced Lilly and other unwed mothers in their maternity decisions. Nevertheless, most participants were determined to assert personal choice and allow less family influence, even if this meant struggling alone. When Marie told her family and friends she was pregnant: ‘there was silence’, followed by little practical support. Individual choices and responsibilities had to be accepted.

The interviewees’ pregnancy decision-making was further complicated by religious moral overtones about sexual behaviour that was reflective of broader Scottish society, despite few interviewees identifying as religious.^69^ Two women with Catholic heritage who were both aged twenty and unmarried when they first fell pregnant in the late 1980s were sent to the Innocence, a Catholic project located in the Govan area of Glasgow that provided medical and social support for women.^70^ Their experiences were very different. Michelle was pressured to put her baby up for adoption but refused, while Sylvia was advised to think about her options. The differing advice may relate to their backgrounds. Michelle had been brought up in care and lacked a supportive family. In contrast, while Sylvia’s parents were upset and her father did not speak to her for 2 weeks, both parents gradually accepted Sylvia’s decision to keep the baby—something which may have been aided by her voluntarily marrying the baby’s father.^71^ Yet despite the pressures surrounding behavioural expectations, some women, including Lilly, Marie, Michelle, and Sylvia, asserted their own choices during pregnancy, while others like Jane struggled against parental control. The interviewees struggled to reconcile individual behavioural choices with moral anxieties over the erosion of community and family values as they navigated the multiple messages of maternity. Nevertheless, the repertoire of standard socially organized ways for reacting to unplanned pregnancies broadened during the latter quarter of the twentieth century from hastily arranged marriages to unmarried motherhood, trial and error cohabitation, and abortion. However, the social stigma associated with pregnancy out of wedlock remained. Not only did these social expectations surrounding behavioural conformity limit reproductive education in schools, they also influenced women’s expectations about antenatal appointments.

## The Complexities within Pregnancy Advice

Under Prime Minister Margaret Thatcher, the College of Health and other groups sought to increase health information for patients, with the aim of empowering patients and transforming the doctor–patient relationship.^72^ Yet, as Mold has argued, the patient consumer of Thatcher’s Britain was a malleable figure, with the rights of individuals gradually superseding the collective needs of patient consumers.^73^ In 1997 the Scottish White Paper *Designed to Care* outlined how a core National Health Service (NHS) objective was ‘providing patients with more information about their health and about their options for treatment…’.^74^ Yet in Glasgow’s low-income neighbourhoods, many patients had limited access to health information and struggled to become an informed consumer. Not only was there a lack of continuity of antenatal provision across the city, but the conservative governments of the 1980s and 1990s blamed the poor for their situation, rather than sympathetically perceiving them as having needs that deserved support from the more fortunate.^75^ The widening social and economic inequalities, combined with limited pregnancy information and the inconsistent provision of maternity care, left the low-income mother unable to become patient consumers.

When asked about their antenatal care choices, few of our Glasgow interviewees remembered having any. Instead, they stressed encounters and locations. One woman recalled first encountering medical practitioners when she arrived at the hospital in labour, having decided that antenatal check-ups were unnecessary.^76^ The other interviewees all attended at least three antenatal appointments, although not always with the same practitioner. When asked which practitioner the women saw when pregnant, one women responded ‘Whoever’s available’, with other participants agreeing ‘Aye. That’s right.’^77^ Similar to Hall, Macintyre, and Porter’s Aberdeen interviewees,^78^ our Glasgow interviewees expressed the desire for consistency of antenatal care provider. Michelle remembered that she ‘felt as if I was juggled, you know, I had nae control over it’.^79^ Yet when she was homeless, she praised the midwives for ‘chasin’ me all over Glasgow’ to monitor her pregnancy. While participants accepted that antenatal care from multiple providers was just ‘the way it was’, several women admitted occasional confusion about appointments and advice.^80^ Participants also admitted having better relations with individual practitioners, although there was no consistency concerning gender or type of practitioner (GP, consultant, or midwife). Liz recalled the midwife for her first baby was ‘auld hatchet face’, whereas for later children she thought ‘the midwife teams are amazin!’. Fiona found her midwife so obnoxious that she ‘would have knocked her head off’,^81^ while Siobhan preferred to see her GP throughout her pregnancy because she had a good relationship with him.^82^ Only women with complex pregnancies were likely to see the same provider, a specialist obstetrician, in hospital. Nevertheless, there was one constant across the focus groups; namely, the desire for sufficient time to ask the practitioner questions and a relaxed atmosphere in which to do so.

Medical encounters were frequently rushed and few women felt comfortable asking questions of their midwife or GP, expressing fear or embarrassment about doing so. When asked if they felt they could ask questions of their caregiver, participants replies included ‘No, I didn’t’, ‘No, it was definitely, you were embarrassed, you know what I mean’, and ‘you were feared to ask them anything when you went’.^83^ Part of the women’s reluctance stemmed from a fear of being judged. While some admitted that having been young mothers might have caused such worries, all interviewees expressed a desire for more information about their pregnancy.^84^ These women did not embrace the ignorance or persona of ignorance about sex and reproduction which Szreter and Fisher found among some women of earlier generations.^85^ Nor did the women find the antenatal period the time when they were educated about pregnancy and childbirth, similar to Angela Davis’s Oxfordshire interviewees.^86^ Rather, most participants agreed with Amy that ‘You had more support [and information] when the baby was born than you got when you were pregnant… Where was Google then, do you know what I mean? It would be so much easier.’^87^ Our interviewees sought knowledge in a culture of silence and judgement.

The broader UK drive to improve antenatal care and pregnancy outcomes after the Second World War and again after the Short Report encouraged healthcare professionals to believe that they were well placed to provide women with the pregnancy information required at antenatal appointments.^88^ In 1991, Geoffrey Chamberlain (then Chairman of the Department of Obstetrics and Gynaecology at St George’s Hospital Medical School, London) argued in the *British Medical Journal* how:

[I]nformal discussions with midwives and doctors at the antenatal clinic are educational and much can be learnt from other mothers in the waiting time at the clinics. This is complemented by many excellent videos…. Many good books exist about pregnancy and childbirth…. A woman should be steered towards a well written account of what she needs in a form that best suits her lifestyle and religious observances and in a language that she can understand.^89^

While such information may have been available, few of our interviewees were the beneficiaries. Any oral and written information provided was minimal (usually taking the form of leaflets), inconsistent or provided in an unhelpful format. Those with literacy challenges would have particularly struggled. While no participant directly revealed their educational attainment, some women required help to complete the background information form. Their educational challenges may have made these women reluctant to ask practitioners questions, seek assistance, or attend antenatal classes. Poor communication, medical jargon, and a lack of information worked to exclude the patient from the medical discourse of pregnancy and the associated technologies. As Jan Williams found in England, women needed to be very secure within their own discourse to ensure that they received the information to enable them to have the pregnancy and birth they desired.^90^ In Glasgow, for women living on low incomes, there was a clear gap between political intent, medical practice, and reality.

Despite lacking the desired pregnancy information and understanding, the women remembered particular people and pieces of advice that broadly followed changing scientific thinking. Several women who experienced their first pregnancy during the 1980s remembered being advised to take iron supplements or to ‘eat black pudding’, whereas in the 1990s women remembered being advised to take folic acid supplements.^91^ Other traditions survived the twentieth century. When pregnant in 1990 with a low iron count, Fiona remembered her midwife advising her to drink a pint of Guinness a day, the rationale being that because she ‘didnae eat red meat….The quick way of getting’ iron into me was a pint o’ Guinness.’^92^ For decades Guinness had been a recommended source of iron for expectant and new mothers, despite it having no higher iron count than other beer, just clever marketing. More commonly, participants remembered advice on healthy eating or how to quit smoking and being offered support groups based at a hospital. Alice chose not to attend such groups, reasoning she ‘wasnae a heavy smoker’.^93^ She was also working and recognized that her job, together with the transportation costs, also contributed to her decision. The lack of community health facilities and the centrality of the hospital for healthcare services suggests patients were expected to ‘fit in with the hospital needs and requirements rather than the other way around’.^94^ While the passage of time can alter memories, the interviewees’ recollections about practitioner encounters suggested their importance as a source of pregnancy information, alongside friends and relations.^95^ Because most interviewees had their first baby during the 1970s through 1990s, they were more dependent on healthcare professionals for antenatal advice than perhaps women with internet literacy are today. Being an informed patient was considered a right which would enable choice, but securing that right remained a struggle.

Support and information for expectant mothers’ stems from many sources, including antenatal classes. Some of Angela Davis’s interviewees from the 1960s through 1980s found antenatal classes helped to dispel women’s ignorance about their bodies, pregnancy, and childbirth, while the friendships made provided an additional source of information and advice. Young mothers, single and working mothers were less likely to attend antenatal classes.^96^ Yet the Glasgow reticence surrounding discussing sex made medical encounters and antenatal classes important sources of pregnancy information. While not all participants revealed whether or not they had attended antenatal classes, well over half admitted either never attending or going only once. The reasons women provided for avoiding antenatal classes included feeling ‘shame’ or ‘embarrassment’ at being poor. They agreed that antenatal classes ‘wasnae for me’.^97^ Other times, work commitments prevented attendance, suggesting the logic central to adaptive decision-making.^98^ Yet those who attended antenatal classes found the information and advice useful. New friendships were not mentioned.^99^ Not only did this lack of regular contact with other pregnant women contribute to social isolation, it left women reliant on ‘clinics and midwives’ and ‘hospitals’, as well as other women for information about impending motherhood.^100^ Yet one group admitted that they did not get their information from female relations.^101^ This class-based gulf between pregnancy experiences and the associated advice and support left our participants feeling that both healthcare professionals and families had left them to ‘get on with it’.^102^ Excluded from the cultural toolkit surrounding pregnancy and motherhood, most of our interviewees felt deprived of the right to be a knowing subject.

## The Enigma Surrounding Maternity Benefits

Despite the lack of ‘formal political autonomy’ prior to devolution in 1997, Scotland had nevertheless developed a ‘distinctive welfare policy’.^103^ Since devolution, the Scottish government has used policy to address certain economic and social peculiarities, including health education. By the early 1990s, ‘health education’ had been largely superseded by ‘health promotion’. Health education emphasized increased individual and community knowledge to enable individuals to manage their own health and had been both a goal of the UK government’s Short Report and the Scottish Office’s first national policy statement dedicated to health in Scotland. In contrast, health promotion comprises ‘social and economic interventions designed to benefit and protect people’s health’.^104^ In Scotland, this left sex education uneven while the political focus emphasized benefits and broader public health campaigns. Yet health and welfare remained largely separate areas of policy and rarely intersected, despite social need. Indeed, the 1999 White Paper Towards a Healthier Scotland emphasized the needs of communities without longer sight of the needs of individuals.^105^ The regular changes in the policy and welfare landscapes of the late twentieth-century distanced individuals living in low-income communities from knowledge about their benefit entitlements.

Although distinct areas of policy, health and welfare are intimately connected. If people are expected to, and indeed wish to, become patient consumers, this involves financial outlay. For pregnant women, this includes everything from travel costs for regular healthcare appointments, to diet, clothing for the expectant mother and newborn, diapers, potential childcare costs for older children, and possibly sterilizing equipment, bottles, and milk. There were also hidden costs of pregnancy, including a charge for an ultrasound picture.^106^ Over the years, different governments have introduced various maternity rights and benefits designed to help pregnant women with costs, time off work for appointments, and maternity pay.^107^ Nevertheless, a woman might be unaware of the full costs of pregnancy and available maternity benefits. Thane and Evans found these challenges were greatest for single mothers, particularly under the conservative governments of the 1980s and 1990s.^108^ Yet in the early twentieth century, women had strong hopes that the introduction of a Universal Maternity Benefit in 1911 would alleviate future women’s financial struggles during pregnancy.^109^ Nearly a century later, our interviewees’ either lacked the awareness of maternity entitlements or faced significant challenges in securing a claim. One interviewee remembered: ‘I don’t know that [maternity benefit], nobody tell me, so I don’t have anything….’ Siobhan agreed: ‘We never got told.’ When she learned of her entitlement to workplace maternity pay, her employer refused. The conflict was only resolved after her baby was born and the Department of Work and Pensions required Siobhan’s employer to pay the maternity benefits owed. Meanwhile, Siobhan had borrowed money from her mother to afford baby supplies and milk.^110^ Borrowing from families to meet immediate financial needs was common practice when welfare benefits did not meet individual needs.

In 1995 over half a million Scots received family credit and over 58 per cent of these had been claiming this benefit for over 2 years. In this vast landscape of Scottish social welfare, benefits were designed to ‘pacify rather than embolden’^111^ the victims of capitalism and circumstance. Yet unknown maternity benefits could do little to pacify the victims. Women learned about entitlements from peer networks, employers, and occasionally healthcare providers. Liz recounted how ‘Ye didnae get a lot o’ help, like, wi’ benefits, you could get, aye… There’s probably that an a’, goin’ back then and even to noo, there’s probably no’ even a’ that information, you know, that somebody should sit you doon and tell you.’^112^ Kirsty, who volunteered at the Citizen’s Advice Bureau, remembered learning about the Blair government’s Sure Start Maternity Grant, a £500 baby premium for women living on a low income, after another pregnant woman requested the relevant information.^113^ Across the focus groups, there was a general frustration that ‘Nobody tells you. And it’s still the same to this day, wi’ any kind, anything that any benefit at all you’re given.’ ‘They don’t tell you.’^114^ Fiona remembered only learning about the Child Tax Credits designed to help offset the costs of raising children when her son was nearly 2 years old.^115^ Child Tax Credits are payable to all primary care givers from a child’s birth until at least aged sixteen, regardless of household income. Women also recalled having been unaware of other maternity benefits, including child benefit and milk tokens.^116^ The Universal Maternity Benefit which had provided hope for the women writing to the Women’s Cooperative Guild in 1915 that their daughter’s pregnancy experiences would be less stressful had only partially materialized.

Particularly unfamiliar to the interviewees were immediate maternity benefits which targeted specific hidden costs, including the reimbursement of travel costs for antenatal appointments. Only one woman remembered being advised about this benefit when, after an ultrasound scan, she was handed a form to complete to reclaim her bus fare.^117^ However, travel costs had to be paid upfront and claimed back, after negotiating the hospital bureaucracy. Most women either paid their own bus fare to appointments or walked.^118^ Transport costs were a predicted cost when labour started. After evaluating taxi fares, many women called an ambulance to take them to the hospital.^119^ Returning home with the newborn proved more challenging and reveals hidden complexities within the welfare system. Most mothers were offered a lift home from the hospital by family or friends or took a taxi. However, Michelle remembered being discharged from the hospital with twins and catching the bus home with her partner because they could neither afford the taxi fare nor the required infant car seats.^120^ These costs fell outside the remit of a medical appointment, while the hospital’s responsibility for the patient ended when the woman walked out the entrance.

The hidden costs of pregnancy quickly added up and women had to balance attending appointments with other expenses and circumstances.^121^ Tension characterized the interviewees’ accounts of negotiating the confusing welfare landscape of late twentieth-century Britain. Single mothers were particularly vulnerable to the financial strains of pregnancy. Some women temporarily moved back in with their mothers for help and support, but an inflexible welfare system meant this could render them ineligible for welfare benefits in their own right.^122^ Yet the mother’s income was frequently insufficient to provide for them both. Single, pregnant women without family support and suffering the eligibility constraints of welfare bureaucracies relied on the voluntary sector and their own initiative. Similar to their 1915 counterparts, in the late twentieth-century women used charities, charity shops, and Glasgow’s Paddy’s market to obtain pushchairs, cots, and other items for baby,^123^ while some received hand-me-downs from friends and relatives. Gender-neutral colours, including lemon, white, or lime, were preferred because they could be reused for any subsequent infants.^124^ While middle-class mothers also made use of hand-me-downs, their wealth offered greater choice about product, colour, and reuse. The overall impression from the interviewees was one of situational awareness, resourcefulness, and determination, something which recent histories of unmarried motherhood, including Kiernan et al. and Thane and Evans have overlooked in favour of policy and provision.^125^ The long-standing NHS commitment to improving maternal and child health retains hidden costs and the Welfare State has some way to go to poverty-proof pregnancy.

## Conclusion

We concluded each interview by asking: ‘What one recommendation would you make to government to improve antenatal provision for all women.’ The women were united with their response, neatly summed up by one: ‘I think they [mothers] need some more information.’^126^ This sentiment clearly highlights participant’s experiences of the health and welfare bureaucracies in contemporary Scotland. It also hints at the cultural context surrounding the broader Scottish reticence towards sex education, particularly between mother and daughter. While changing health and welfare policies increased support and healthcare options for all pregnant women, participants highlighted the continuing guilt and responsibility surrounding being an unwed mother or being poor and pregnant. In late twentieth-century Scotland, being poor and pregnant required reconciling values and realities, traditions and modernity. While maternity remained valued in the early 2000s, maternity had changed. The traditional values invested in it were only occasionally realized. Maternity healthcare and welfare had also changed, but our interviews suggest the confusion and class-based biases surrounding provision and health and welfare information remained.

Low-income expectant mothers could rarely be the informed patient–consumer or consumer–patient that the conservative governments constructed in the late twentieth century. As Mold highlighted, this was the preserve of certain primarily middle-class interests. Instead, the Glasgow participants in this study reacted to their immediate situation and daily balanced healthcare, expenditure, work, and maternity. Adaptive decision-making enabled each woman to deal with each situation she faced in turn and in the manner she thought best for herself as an individual, an expectant mother, and as a woman. On their rocky road, motherhood and womanhood merged only sometimes. At other times, socio-economic practicalities and the moral dynamics of modern Scotland separated these and fractured their meanings.

## Footnotes

1 G. Bramely, et al., ‘Benefit Take-Up and the Geography of Poverty in Scotland’, *Regional Studies*, 34 (2000), 516; Catriona Macdonald, *Whaur Extremes Meet: Scotland’s Twentieth Century* (Edinburgh, 2009), 138.

2 D. Walsh, et al., ‘”It’s Not Just Deprivation”: Why Do Equally Deprived UK Cities Experience Different Health Outcomes?’, *Greater Glasgow Population Health* (September 2010), 1–12; M. Reid, ‘Behind the “Glasgow effect”’, *Bulletin of the World Health Organization*, 89 (2011), 706–7; Simon Fraser and Steve George, ‘Perspectives on Differing Health Outcomes by City: Accounting for Glasgow’s Excess Mortality’, *Risk Management and Health-Care Policy*, 8 (2015), 99–110.

3 L. Bryder and J. Greenlees, eds, ‘Western Maternity and Medicine: An Introduction’, in J. Greenlees and L. Bryder, eds, *Western Maternity and Medicine, 1880-1990* (London, 2013), 3–5.

4 Ann Oakley, *Women Confined: Towards a Sociology of Childbirth* (New York, 1988), 20.

5 A. Davis, *Modern Motherhood: Women and Family in England, 1945-2000* (Manchester, 2012), 87–88; M. Hall, et al., *Antenatal Care Assessed: A Case Study of an Innovation in Aberdeen* (Aberdeen, 1985), 1–2.

6 Oakley, *Women Confined*, 20.

7 J. L. Emery, ‘Perinatal Mortality and Morbidity: Discussion of the “Short Report”’, *Journal of the Royal Society of Medicine*, 74 (1981), 934.

8 Short Report: Second Report from the Social Services Committee. *Perinatal and Neonatal Mortality* (London, 1980), 127–8; Scottish Health Education Group, *The Book of the Child* (Edinburgh, 1980).

9 e.g. J. J. Perkins and R. W. Sanson-Fisher, ‘Increased Focus on the Teaching of Interactional Skills to Medical Practitioners’, *Advances in Health Sciences Education*, 1 (1996), 17–28; A. Donabedian, ‘The Quality of Care’, *Journal of the American Medical Association*, 260 (1988), 1743–8; C. Helman, ‘Communication in Primary Care: The Role of Patient and Practitioner Explanatory Models’, *Social Science Medicine*, 20 (1985), 923–31; H. Weschler, et al., ‘The Physician's Role in Health Promotion — A Survey of Primary Care Practitioners’, *New England Journal of Medicine*, 308 (1983), 97–100; D. J. Cegala and S. Lenzmeier Broz, ‘Physician Communication Skills Training: A Review of Theoretical Backgrounds, Objectives and Skills’, *Medical Education*, 36 (2002), 1004–16.

10 Hall, *et**al., Antenatal Care*, 24.

11 Lindsay Reid, *Midwifery in Scotland: A History* (Erskine, 2011), 91–92.

12 A. Mold, ‘Repositioning the Patient: Patient Organization, Consumerism and Autonomy in Britain during the 1960s and 1970s’, *Bulletin of the History of Medicine* 87 (2013), 227; A. Mold, ‘Making the Patient-Consumer in Margaret Thatcher’s Britain’, *The Historical Journal*, 54 (2011), 509–28.

13 Anna Dixon and Julian Le Grand, ‘Is Greater Patient Choice Consistent with Equity? The Case of the English NHS’, *Journal of Health Services Research and Policy*, 11 (2006), 162–6.

14 M. Hilton, ‘The Female Consumer and the Politics of Consumption in Twentieth-Century Britain’, *The Historical Journal*, 45 (2002), 103–28.

15 P. Matthews and A. Hastings, ‘Middle-Class Political Activism and Middle-Class Advantage in Relation to Public Services: A Realist Synthesis of the Evidence Base’, *Social Policy and Administration*, 47 (2013), 72–92.

16 Bryder and Greenlees, ‘Western Maternity’, 1–12.

17 Ann Oakley, *The Captured Womb: A History of the Medical Care of Pregnant Women* (Oxford, 1984).

18 e.g. Salim Al-Gailani, ‘Pregnancy, Pathology and Public Morals: Making Antenatal Care in Edinburgh around 1900’, in Greenlees and Bryder, eds, *Western Maternity*, 31–46; Marjory Tew, *Safer Childbirth? A Critical History of Maternity Care* (London, 1998); Judith Walzer Leavitt, *Brought to Bed: Childbearing in America 1750-1950* (Oxford, 1986).

19 e.g. Jean Donnison, *Midwives & Medical Men: The History of the Struggle for the Control of Childbirth* (Whitstable, 1988); Tew, *Safer Childbirth.*

20 V. Fildes, et al., eds, *Women and Children First: International Maternal and Infant Welfare 1870-1945* (London, 1992); L. Marks, *Metropolitan Maternity: Maternal and Infant Welfare Services in Early Twentieth Century London* (Amsterdam, 1996); A. Susan Williams, *Women & Childbirth in the Twentieth Century: A History of the National Birthday Trust Fund 1928-93* (Stroud, 1997).

21 L. Earner-Byrne, ‘Twixt God and Geography: The Development of Maternity Services in Twentieth-Century Ireland’, in Greenlees and Bryder, eds, *Western Maternity*, 99–112.

22 Oakley, *Captured Womb;* Oakley, *Women Confined;* J. Lewis, *The Politics of Motherhood: Child and Maternal Welfare in England, 1900-1939* (London, 1980)*;* Jan Williams, ‘The Controlling Power of Childbirth in Britain’, in H. Marland and A. M. Rafferty, *Midwives, Society and Childbirth* (London, 1997), 232–47; Davis, *Modern Motherhood*; A. Nuttall, ‘Taking “Advantage of the Facilities and Comforts … Offered”: Women’s Choice of Hospital Delivery in Interwar Edinburgh’, in Greenlees and Bryder, eds, *Western Maternity*, 65–80.

23 e.g. the primarily state-sponsored early twentieth-century infant and maternal welfare movement. A. Davin, ‘Imperialism and Motherhood’, *History Workshop Journal*, 5 (1978), 9–66; D. Dwork, *War**Is Good for Babies and Other Young Children: A History of the Infant and Child Welfare Movement in England* (London, 1987); C. Dyhouse, ‘Working-Class Mothers and Infant Mortality in England, 1895-1914’, *Journal of Social History*, 12 (1978), 248–66; Lewis, *Politics of Motherhood*; Marks, *Metropolitan Maternity*.

24 Davis, *Modern Motherhood;* Jo Murphy Lawless, *Reading Birth and Death: A History of Obstetrical Thinking* (Bloomington, 1998), 22.

25 Matthews and Hastings, ‘Middle-Class’, 85.

26 Hilary Graham and Lorna McKee, *The First Months of Motherhood Volume I: Summary Report*, unpublished report (London, 1979); Sally Macintyre, ‘Expectations and Experiences of First Pregnancy’, *Occasional Paper no. 5* (University of Aberdeen, 1981), cited in Oakley, *Captured Womb*, 245; Maureen Porter and Sally Macintyre, ‘Psychosocial Effectiveness of Antenatal and Postnatal Care’, in S. Robinson and A. M. Thomson, eds, *Midwives, Research and Childbirth* (Boston, 1989), 72–94.

27 H. Gross and H. Pattison, ‘Pregnancy and Working: A Critical Reading of Advice and Information on Pregnancy and Employment’, *Feminism and Psychology*, 11 (2001), 515.

28 Davis, *Modern Motherhood*, 91.

29 Elizabeth Roberts, *A Woman’s Place: An Oral History of Working-Class Women, 1890-1940* (Oxford, 1984), 81–109; Elizabeth Roberts, *Women and Families: An Oral History, 1940-1970* (Oxford, 1995), 59–72, 76–80; Lucinda Beier, *For Their Own Good: The Transformation of English Working-Class Health Culture, 1880-1970* (Columbus, 2016); Kate Fisher, *Birth Control, Sex and Marriage in Britain, 1918-1960* (Oxford, 2006); Simon Szreter and Kate Fisher, *Sex before the Sexual Revolution: Intimate Life in England, 1918-1963* (Cambridge, 2010).

30 Pat Thane and Tanya Evans, *Sinners? Scroungers? Saints?: Unmarried Motherhood in Twentieth-Century England* (Oxford, 2013).

31 Margaret Llewelyn Davies, ed., *Maternity: Letters from Working Women* (London, 1915; 1978 ed.).

32 e.g. L. Abrams, *Glasgow: High-Rise Homes, Estates and Communities in the Post-War Period* (London, 2020); L. Jamieson, ‘Changing Intimacy: Seeking and Forming Couple Relationships’, in L. Abrams and C. Brown, eds, *A History of Everyday Life in Twentieth-Century Scotland* (Edinburgh, 2010), 76–102; A. McIvor, ‘The Realities and Narratives of Paid Work: The Scottish Workplace’, in Abrams and Brown, eds, *History of Everyday Life*, 103–30.

33 <http://www.povertyalliance.org/> accessed 9 March 2021. Participants gave permission for their names to be used. Pseudonyms were adopted to protect privacy and in case a participant later changes her mind.

34 C. Moser and G. Calton, *Survey Methods in Social Investigation* (Abingdon, 1971; 2016 ed.), 204–5.

35 e.g. Beier, *For Their Own Good*, 245–53; Fisher, *Birth Control*, 60–65, 113–29; Francesca Moore, ‘Beyond the Ideal: Motherhood in Industrial Lancashire, 1860-1937’, PhD thesis, University of Cambridge, 2008; Janet Greenlees, ‘Workplace Health and Gender among Cotton Workers in America and Britain, c. 1880s-1940s’, *International Review of Social History*, 61 (2016), 459–85; Roberts, *Women and Families.*

36 Alex Mold, *Making the Patient-Consumer: Patient Organisations and Health Consumerism in Britain* (Manchester, 2015), 117–207.

37 As cited in Jane Lewis and Trudie Knijn, ‘The Politics of Sex Education in England and Wales and the Netherlands since the 1980s’, *Journal of Social Policy*, 31 (2002), 685.

38 Kate Fisher, ‘“She Was Quite Satisfied with the Arrangements I Made”: Gender and Birth Control in Britain, 1920-1950’, *Past and Present*, 169 (2000), 189.

39 Beier, *For**Their Own Good*, 236–9; Szreter and Fisher, *Sex*, 55.

40 Szreter and Fisher, *Sex*, 63–110; 96, 99; Selina Todd, *Young Women, Work and Family in England, 1918-1950* (Oxford, 2005), 155–6.

41 Lesley Hall, ‘Birds, Bees and General Embarrassment: Sex Education in Britain, from Social Purity to Section 28’, in Richard Aldrich, ed., *Public or Private Education?: Lessons from History* (London, 2004), 98.

42 Roger Davidson, *Dangerous Liaisons: A Social History of Venereal Disease in Twentieth-Century Scotland* (Amsterdam, 2000), 293; General Assembly of the Church of Scotland (GACS) Report of the Moral Welfare Committee, 1977, 305–6; Also cited in R. Davidson and G. Davis, *The Sexual State: Sexuality and Scottish Governance, 1950-1980* (Edinburgh, 2012), 205.

43 Davidson, *Dangerous Liaisons*, 293.

44 Davidson and Davis, *Sexual State*, 205.

45 Roger Davidson and Gayle Davis, ‘This Thorniest of Problems: School Sex Education Policy in Scotland, 1939-80’, *Scottish Historical Review*, 84 (2005), 243.

46 Davidson and Davis, ‘This Thorniest of Problems’, 298.

47 Davidson and Davis, ‘This Thorniest of Problems’, 295.

48 Davidson and Davis, *Sexual State*, 295–6.

49 Davidson and Davis, *Sexual State*, 207.

50 Davidson and Davis, ‘This Thorniest of Problems’, 206; Davis, *Modern Motherhood*, 60; John T. Smith, *Key Questions in Education: Historical and Contemporary Perspectives* (London, 2016), Chapter 9.

51 Davis, *Modern Motherhood*, 63–65.

52 House of Commons Social Service Committee, Problems Associated with AIDS, 171; cited in Davidson and Davis, *Sexual State*, 207; my emphasis.

53
*The Scotsman*, 7 January 2017.

54 Maryhill, 22 March 2017.

55 Priesthill, 28 August 2017.

56 Priesthill, 28 August 2017.

57 Maryhill, 22 March 2017. These responses reflect Jamieson’s findings for working-class urban Scotland. Jamieson, ‘Seeking and Forming’, 85; Sally Macintyre, *Single and Pregnant* (London, 1977).

58 Llewelyn Davies, ed., *Maternity*, e.g. 30, 33, 48, 50, 56, 59, 72, and 81; Fisher, ‘“She Was Quite Satisfied”; Davis, *Modern Motherhood*, 56–78. The desire for reproductive information should not be conflated with ignorance and repression in sexual cultures. Szreter and Fisher, *Sex*.

59 Lucinda McCray Beier, ‘“We Were Green as Grass”: Learning about Sex and Reproduction in Three Working-Class Lancashire Communities, 1900-1970’, *Social History of Medicine*, 16 (2003), 475; Szreter and Fisher, *Sex.*

60 A. Blaikie, ‘”A Kind of Loving”: Illegitimacy, Grandparents and the Rural Economy of Northeast Scotland, 1750-1900’, *Scottish Economic and Social History*, 14 (1994), 41–57; A. Blaikie, ‘Scottish Illegitimacy: Social Adjustment or Moral Economy’, *Journal of Interdisciplinary History*, 29 (1998), 221–41.

61 Maryhill, 26 July 2017.

62 Amy Schalet, ‘Raging Hormones, Regulated Love: Adolescent Sexuality and the Construction of the Modern Individual in the US and the Netherlands’, *Body and Society*, 6 (2000), 75–105.

63 Jamieson, ‘Seeking’, 93–94.

64 Maryhill, 22 March 2017.

65 K. Hinds and L. Jamieson, ‘Rejecting Traditional Family Building?: Attitudes to Cohabitation and Teenage Pregnancy in Scotland’, in J. Curtice, et al., eds, *New Scotland, New Society* (Edinburgh, 2001), 37, 38, 40.

66 Maryhill, 22 March 2017.

67 Emily Robinson, et al., ‘Telling Stories about Post-War Britain: Popular Individualism and the “Crisis” of the 1970s’, *Twentieth Century British History*, 8 (2017), 273–4.

68 Robinson, et al., ‘Telling Stories about Post-War Britain’, 272.

69 Callum Brown, *Religion and Society in Scotland since 1707* (Edinburgh, 1997), 158–61, 170–1.

70 The Innocence Project has closed.

71 Pollock, 6 November 2017.

72 Mold, ‘Making the Patient-Consumer’, 518, 528.

73 Mold, ‘Making the Patient-Consumer’, 528.

74 Scottish Government, *Designed to Care: Renewing the National Health Service in Scotland*, Scottish Office White Paper, Cm. 3811, 8 December 1997, 14.

75 Similar to Thane and Evans’ findings for unwed teenage mothers in England. Thane and Evans, *Sinners*, Chapter 8, esp. 169.

76 Maryhill, 22 March 2017.

77 Maryhill, 22 March 2017.

78 Hall, et al., *Antenatal Care*, 89.

79 Pollock, 6 November 2017.

80 Priesthill, 28 August 2017.

81 Priesthill, 28 August 2017.

82 Maryhill, 26 July 2017.

83 Maryhill, 26 July 2017; Priesthill, 28 August 2017.

84 Priesthill, 28 August 2017; Maryhill, 22 March 2017 and 26 July 2017.

85 Szreter and Fisher, *Sex*, 63–110.

86 Davis, *Modern Motherhood*, Chapter 3, esp. 78.

87 Maryhill, 22 March 2017.

88 Geoffrey Chamberlain, ‘ABC of Antenatal Care: Normal Antenatal Management’, *BMJ*, 302 (30 March 1991), 774–9; Jean Grime, ‘The Maternity Patient in Hospital: An Experiment in Health Education’, *Nursing Times*, 61 (1965), 249–50; P. S. Cook, ‘Antenatal Education for Parenthood, as an Aspect of Preventive Psychiatry: Some Suggestions for Programme Content and Objective’*, Medical Journal of Australia*, 13 (1970), 676–81*.*

89 Chamberlain, ‘ABC’, 779.

90 Williams, ‘Controlling Power’.

91 Maryhill, 22 March 2017; Pollock, 6 November 2017.

92 Priesthill, 28 August 2017.

93 Priesthill, 28 August 2017.

94 Davis, *Modern Motherhood*, 91.

95 Maryhill, 22 March 2017.

96 Davis, *Modern Motherhood*, 70–77.

97 Maryhill, 26 July 2017; Priesthill, 28 August 2017.

98 Priesthill, 28 August 2017; Pollock, 6 November 2017.

99 Maryhill, 22 March 2017.

100 Maryhill, 26 July 2017.

101 Maryhill, 22 March and 26 July 2017.

102 Maryhill, 22 March and 26 July 2017; Priesthill, 28 August 2017; Pollock, 6 November 2017.

103 R. Lowe, *The Welfare State in Britain since 1945* (Basingstoke, 2005), 95.

104 WHO, ‘Health Promotion’, 2016 <https://www.who.int/news-room/questions-and-answers/item/health-promotion> accessed 11 January 2022.

105 Andrew Tannehill, ‘Promoting Health in Scotland’, in K. Woods and D. Carter, eds, *Scotland’s Health and Health Service* (London, 2003), 183.

106 These charges were removed from Scottish health boards in 2017. *The Herald*, ‘Call to Stop Charging Highland Mothers £5 for Baby Scan’ <https://www.heraldscotland.com/news/health/15416205.call-to-stop-charging-highland-mothers-5-for-baby-scan-photo/> accessed 20 December 2018.

107 From 1994, all women had the right to a minimum of 14 weeks’ maternity leave with a right to return to work, irrespective of the hours worked or length of service. Women who had been continuously employed for 26 weeks were eligible for statutory maternity pay which for the first 6 weeks was 90 per cent of earnings and £52.50 for the remaining 12 weeks. The Trade Union Reform and Employment Rights Act, 1993. Claire Callender, ‘Women and Employment’, in Christine Hallett, ed., *Women and Social Policy: An Introduction* (London, 1996), 44.

108 Thane and Evans, *Sinners?*, 174–8.

109 Llewelyn Davies, ed., *Maternity*, e.g. 131, 72–3, 105, 138–40.

110 Maryhill, 22 March 2017.

111 Bramely, et al., ‘Benefit’, 516; Scottish Abstract of Statistics, 25 (HMSO, 1996), 45; Macdonald, *Whaur Extremes Meet*, 137–8.

112 Priesthill, 28 August 2017.

113 In December 2018, the Pregnancy and Baby Payment replaced the Sure Start Maternity Grant, expanding benefits and extending eligibility.

114 Priesthill, 28 August 2017.

115 Priesthill, 28 August 2017.

116 Intended for baby milk, milk tokens could be used for fruit and vegetables. *Pollock*, 6 November 2017.

117 This practice remains today but the process causes confusion, especially when a hospital lacks cashier facilities. The patient must then mail the form with travel receipts within 3 months of her appointment.

118 Maryhill, 22 March 2017.

119 Maryhill, 26 July 2017.

120 Pollock, 6 November 2017.

121 A roundtable between the author and Glasgow midwives and physiotherapists in November 2018 suggests this remains the case.

122 Maryhill, 26 July 2017.

123 Until its closure in 2009, Paddy’s Market provided a lifeline for Glasgow’s poor where they could purchase second-hand goods.

124 Maryhill, 26 July 2017.

125 K. Kiernan, et al., *Lone Motherhood in Twentieth-Century Britain* (Oxford, 1998); Thane and Evans, *Sinners?*

126 Maryhill, 22 March 2017.

## Notes

My thanks to the Wellcome Trust for their generous funding of this research. I would also like to thank John Stewart, members of the Reproduction Virtual Circle and the reviewers for their helpful suggestions on previous versions of this article. I also benefitted from audience feedback to associated presentations at the AAHM and EAHMH 2017 and the CAHNH 2018. This research was funded in whole, or in part, by the Wellcome Trust Grant No. 205650/Z/16/Z. For the purpose of Open Access, the author has applied a CC BY public copyright license to any Author Accepted Manuscript version arising from this submission.

